# Environmental Risk and Credit Ratings, and the Moderating Effect of Market Competition

**DOI:** 10.3390/ijerph19095341

**Published:** 2022-04-27

**Authors:** Yujin Kim, Su-In Kim

**Affiliations:** 1 Hana Institute of Finance, Hana Bank, Seoul 04538, Korea; jazzgene@naver.com; 2 College of Business Management, Hongik University, Sejong 30016, Korea

**Keywords:** environmental risk, credit ratings, market competition, *HHI* (Herfindahl-Hirschman Index)

## Abstract

This study examines the relationship between environmental risk and corporate bond credit ratings, and the moderating effect of market competition. We focus on Korean firms that are facing increasing risk of environmental crisis after the COVID-19 pandemic. Recently, the Korean government has been controlling businesses while promoting policies to transform the economy into a low-energy, low-carbon economy. We find that a firm’s greenhouse gas emission and energy consumption, which are direct indicators of environmental risk, are negatively associated with bond credit ratings. We also report that the negative effect of environmental risk on credit ratings is stronger in firms with low market competition. This study contributes to prior research by improving the understanding of the effect of environmental risk on credit ratings. In particular, it is significant to examine the effect of environmental risk, measured as direct environmental performance not affected by green washing, on credit rating. Therefore, we shed light on environment-oriented management beyond the determinants of credit ratings, which have been discussed in previous studies. We also suggest that policymakers need to manage market competition in terms of environmental justice, given that market competition has a significant moderating effect on the relationship between environmental risk and credit ratings.

## 1. Introduction

The whole world is fighting the unprecedented COVID-19 pandemic, and interest in climate change and environmental protection is growing rapidly. The belief that the rapid increase in the frequency of abnormal climate conditions (forest fires, drought, flooding, etc.) caused by environmental destruction is the cause of infectious diseases such as COVID-19 is gaining ground [1]. The ‘Global Risk Report 2021’ published by the World Economic Forum mentions extreme weather, climate action failure, human environmental damage, infectious diseases, and biodiversity loss as the top five risk factors most likely to be faced by humanity.

In light of these observations, interest in environmental issues around the world is expected to increase, especially due to the establishment of the Joe Biden government in the United States, which emphasizes eco-friendly policies and fully supports the EU Green Deal that was implemented in 2021. In particular, major countries believe that the provision of sustainable energy resources, such as renewable energy, helps to find sources for sustainable economic growth [2]. That is, it is important to note that a key principle of the global energy security system is the rational use of traditional energy resources [3,4].

Korea has high potential for carbon leakage because its main industries are high-carbon emitting industries, such as steel and petrochemicals, and the level of coal power generation as an energy source is high. Korea’s carbon dioxide emissions are the ninth highest in the world (as of 2019), and the increase in carbon emissions is the highest among OECD countries [5].

Accordingly, the Korean government is implementing policies to transform the economy into a low-energy, low-carbon economy through the development of greenhouse gas reduction technologies and the initiation of green infrastructure investment. Following the Korean New Deal in July 2020, the Korean government declared ‘2050 carbon neutrality’ as one of its primary long-term goals, along with three other major environmental policies in December 2020: low carbonization of the economic structure, creation of an ecosystem for a new promising low-carbon industry, and fair transition to a carbon-neutral society [6].

Specifically, the government is expanding the regulations on corporate’ greenhouse gas emissions through the Greenhouse Gas Emissions Trading Scheme’ and the Management of Targets for Greenhouse Gas and Energy. The Korean government introduced and implemented the ‘Management of Targets for Greenhouse Gas and Energy’ in response to climate change and managing energy goals in 2010. Some of the greenhouse gas emission permits must be purchased (allocated for a fee) from the government through an auction. They were allocated free of charge during the initial period of 2015–2017. However, from 2018 to 2020, 3% were allocated for a fee, and from 2021, the allocation ratio will increase to 10%. As the government’s greenhouse gas reduction target is upwardly adjusted, environmental regulations are becoming an important factor affecting the corporate business environment.

Considering the increasing environmental costs of firms, environmental, social, and governance (ESG) activities, including environmental justice, are used as key indicators to evaluate a company’s creditworthiness in the capital market. Here, ESG, which consists of the non-financial environmental, social, and governance elements, is a philosophy for corporate sustainability. Recently, this concept has emerged as a keyword that determines the success or failure of capital market and the nation beyond the firm level. Focusing on Korean firms that urgently respond to environmental risks, this study examined the effects of corporate environmental risks (energy consumption and carbon emission) on credit ratings and the moderating effect of market competition.

As environmental and carbon-related legislation tightens, capital market participants are increasingly incorporating environmental issues into their decisions [7,8,9,10,11]. Environmental performance, such as high CO₂ emissions, has a negative effect on financial performance [12,13,14,15,16]. Thus, a firm’s exposure to environmental risk increases the uncertainty inherent in its current and future cash flows, and, ultimately, the probability of default [17,18,19]. This is because firms with high environmental risk are more likely to be exposed to uncertainties related to future climate change risks. Based on the above discussion, we expect that environmental risks, such as greenhouse gas emissions and energy consumption, negatively affect the corporate bond credit ratings.

Previous studies on market competition and corporate performance report that the greater the competition intensity, the higher the productivity and efficiency of the firm [20,21]. This is because the agency problem between the manager and the shareholder decreases, as the private utility of the majority shareholder and the manager’s hazard decrease in highly competitive firms [22,23,24]. The competitive environment acts as external governance of a firm, thereby promoting efficient management [21,22,25,26] and, in turn, reducing the risk premium in the capital market [27].

Therefore, the lower the competition intensity, the lower the company’s credit rating [28], and further, the negative effect between environmental risk and credit rating may become stronger. This is because, if competition, which plays a role in governance, is weak, environmental risks that negatively affect the firm value may increase. Based on the above discussion, the negative relationship between environmental risk and corporate bond credit ratings is expected to be stronger in firms with low market competition.

From the analysis of a sample of firms listed on the Korea Stock Exchange (KSE) and Korean Securities Dealers Automated Quotations (KOSDAQ) from 2011 to 2019, we find that there is a negative relationship between environmental risk and corporate bond credit ratings.

We find that a firm’s greenhouse gas emissions and energy consumption, which are direct indicators of environmental risk, are negatively associated with bond credit ratings. Thus, we can infer that credit rating agencies (CRAs) may recognize a firm’s environmental risk as a negative factor when evaluating credit ratings. This result shows the effect of environmental risk, measured as direct environmental performance not affected by greenwashing, on credit rating. We also report that the negative effect of environmental risk on corporate bond credit ratings is stronger in firms with low market competition, which suggests that market competition has an important moderating effect on the relationship between environmental risk and credit ratings.

This study contributes to prior research on credit ratings by improving the understanding of the effect of environmental risk on corporate bond ratings, using the actual greenhouse gas emissions and energy consumption of Korean firms. Therefore, we provide a convincing answer to the puzzle in environmental management beyond the determinants of credit rating (i.e., accounting information, governance, etc.), which have been discussed in previous studies. We suggest that policymakers need to manage market competition in terms of environmental justice, given that market competition has a significant moderating effect on the relationship between environmental risk and credit ratings.

The remainder of this paper is organized as follows. In Section 2, we review the related literature on the determinants of credit ratings and the effect of environmental performance on financial performance, and develop the hypothesis. We describe the research design, including sample selection and data collection, in Section 3, and the empirical results are presented in Section 4. Finally, we conclude the paper by discussing the implications of the results in Section 5.

## 2. Literature Review and Hypothesis Development

### 2.1. Determinants of Credit Ratings

A credit rating is an index that represents the credit risk (default risk) of a firm (the debtor) or a specific debt (corporate bond) and is a criterion for determining the expenses (interest rate) incurred when using capital from others in the market. The purpose of a credit rating is to communicate information about possible debt redemption risks to investors. Therefore, firms must attach credit rating information, as assessed by credit rating agencies (CRAs), when they raise long-term funds directly from the financial market by issuing corporate bonds. Such credit rating information, which is assessed and disclosed through a public authority, is responsible for reducing information asymmetry in the bond market by providing investors with objective and reliable information about the firms and inducing efficient allocation of resources within the capital market [29]. In Korea, to raise funds by issuing bonds, a company must designate two or more credit rating agencies to provide credit ratings. The three major credit rating agencies in Korea evaluate a company’s credit risk as AAA, AA, A, BBB, BB, B, CCC, CC, C, or D, and related information is available on the websites of the Financial Supervisory Service, Korea Exchange, Korea Financial Investment Association, and Korea Investors Service.

Risk factors considered by CRAs when assessing credit ratings consist of management risk, affiliate risk, industry risk, business risk, and financial risk [30]. Management risk is the credit risk to the operating entity of the firm, which is related to the quality and predictability of management, the TMT’s intention, and the management plan and strategy. Specifically, CRAs review the equity and governance structure, internal control system, management capability and propensity, corporate culture, management policy, and management strategy, and other management specifics, to assess management risk.

Recently, as corporate legal and social interest in ESG management has expanded, it has become more important to consider ESG activities in management risk. Since the United Nations Principles of Responsible Investment (UN PRI) resolution of 2006 emphasized corporate responsibility for environmental, social, and governance factors, ESG has been taken into account when evaluating companies in the capital markets. In other words, the main means of signaling a company’s internal information to the capital market is expanding from financial information (dividend policy, stock split, treasury stock acquisition, etc.) to non-financial information. Quantitative information based on financial information is easy to objectify, but because of its limited scope, there are limits to credit evaluation and prediction of corporate bankruptcy.

According to previous studies examining the relationship between ESG factors and credit ratings, disclosure of ESG activities by companies, such as publication of sustainability reports, reduces information asymmetry between companies and investors, and consequently reduces uncertainty in default. Polbennikov et al. [31] studied the historical relationship between ESG ratings and corporate bond spreads and reported that corporate bonds with high ESG ratings have lower spreads. Kiesel and Lücke [32] examined environmental, social, and governance considerations in rating reports published by CRAs and suggested that ESG consideration is a significant factor in the stock return and CDS spread around the time of the rating announcement. Jang et al. [33] analyzed the relationship between ESG scores and bond returns and found that high environmental scores lower the cost of debt financing, especially for small firms. Park and Noh [18] reported a negative relationship between climate change risk and the cost of capital measured by the weighted average cost of capital. They also found that companies with high levels of greenhouse gas emissions or energy use are more likely to be exposed to the uncertainty related to future climate change risks. Lemma et al. [19] analyzed the relationship between corporate carbon risk, voluntary disclosure, and cost of capital for South Africa, which is a “rising power” in the climate policy debate. They suggested that firms could take advantage of voluntary carbon disclosure to reduce the cost of capital.

In addition to the reporting in previous studies that CSR has a positive effect on credit ratings, it is also notable that the credit rating, which evaluates the repayment possibility of principal and interest, is a different concept from the ESG rating (Korea Investors Service). Korea Investors Service (KIS) is a credit rating agency affiliated with Moody’s Investors Service, and is one of the three largest CRAs (Korea Investors Service, NICE Investors Service, Korea Ratings) in Korea. In the credit rating process, CRAs include ESG factors as other factors to consider for deriving firms’ stand-alone ratings after model evaluation based on management risk, affiliate risk, industry risk, business risk, and financial risk. However, ESG factors do not directly or strongly affect corporate credit ratings, and it is difficult to isolate how much ESG is actually reflected in the final credit rating (Korea Investors Service).

### 2.2. The Effect of Environmental Performance on Financial Performance

Numerous previous studies have reported that environmental performance (i.e., CO₂ emissions, etc.) has a negative effect on financial performance from a long-term perspective. Russo and Fouts [12] reported that, regarding the resource-based view of the firm, environmental performance and economic performance have a positive relationship, with higher returns from environmental performance, especially in high-growth industries. Konak and Cohen [13] found that poor environmental performance has a negative effect on the value of intangible assets in the S&P 500 firms. Saka and Oshika [14] reported that CO₂ emissions have a negative effect on firm value, after taking into account net assets, earnings before extraordinary items, and earnings forecasts, and disclosure on CO₂ alleviates the negative effect on the firm value.

Matsumura et al. [15] clarified that, for every thousand metric tons of carbon emissions added, the firm value decreases on average by USD 212,000, using carbon emissions data that were voluntarily disclosed by S&P 500 firms to the Carbon Disclosure Project. Busch and Lewandowski [16] report that corporate carbon performance is negatively related to both accounting-based and market-based financial performance. In addition, studies have reported that environmental risks increase the cost of capital. Jung et al. [34] investigated whether lenders take a firm’s exposure to carbon risk into account in their lending decisions, and documented a positive relationship between the carbon risk and cost of debt.

### 2.3. Hypotheses Setting

As environmental and carbon-related legislation tightens, capital market participants (such as lenders, investors) are increasingly incorporating environmental risk into decisions (such as lending, investment) [7,8,9,10,11]. Previous studies suggested that environmental performance (i.e., CO₂ emissions, etc.) has a negative effect on financial performance [12,13,14,15,16]. Based on this, we can see that firms’ exposure to environmental risk adds to the uncertainty inherent in current and future cash flows, and ultimately increases the probability of default. As a result, funders (lenders or investors) are more likely to impose a higher risk premium on firms with higher environmental risk [35,36]. Therefore, we expect that credit rating agencies perceive environmental risks, such as greenhouse gas emissions and energy consumption, as a negative factor in terms of management risk. Thus, the following hypothesis is established:

**Hypothesis** **1** **(H1).** *Environmental risk harms corporate bond credit ratings.*

Previous studies on market competition and corporate performance report that the greater the competition intensity, the higher the productivity and efficiency of the firm [20,21]. As the private utility of the majority shareholder and the manager’s hazard decreases in highly competitive firms, the agency problem between the manager and the shareholder decreases [22,23,24].

Griffith [20] analyzed the relationship between product market competition, and productivity levels and growth rates, using panel data on UK establishments, and reported that increased product market competition due to the Single Market Program (SMP) resulted in improved efficiency levels and growth rates. Baggs and Bettignies [21] found that competition has a significant impact on contractual incentives and employee effort, leading to quality improvements and cost savings in Canada. Defond and Park [23] reported that the frequency of CEO turnover is higher in highly competitive industries than in less competitive industries, as competition enhances the usefulness of relative performance evaluation (RPE), which improves boards’ ability to identify unfit CEOs. Hart [22] argued that competition in the product market reduces managerial slack.

As discussed above, a competitive environment acts as a form of external governance of a firm, thereby reducing agency costs and promoting efficient management [21,22,25,26] and, in turn, reducing the risk premium in the capital market [27]. Therefore, the lower the competition intensity, the lower the company’s credit rating [28], and further, the negative effect between environmental risk and credit rating may become stronger. This is because, if competition, which plays a role as governance, is weak, environmental risks that negatively affect firm value may increase. In other words, we expect that the negative evaluation of environmental risk will be more prominent in firms with low competition in the credit rating evaluation of credit rating agencies; thus, Hypothesis 2 is established:

**Hypothesis** **2** **(H2).** *The negative relationship between environmental risk and corporate bond credit ratings is expected to be stronger at firms with low market competition.*

## 3. Materials and Methods

### 3.1. Model

We developed Equation (1) to test Hypothesis 1. An ordered logit regression model was designed with the credit rating score as the dependent variable and the factors affecting the credit rating as the independent variable, referring to previous studies [37]. In addition, to avoid endogeneity problems that may occur between the dependent variable and the independent variable, a lag is set for one accounting period.

The dependent variable, credit rating (*GRADE*), is measured by scoring the credit ratings of domestic credit rating agencies. We give points at equal intervals in descending order from the highest AAA grade to the lowest D grade. As a result, the highest value for the grade is 20, and the lowest is 1.

Environmental risk (*ER*), the independent variable, is measured in a firm’s greenhouse gas emissions and energy consumption. *ER* consists of four variables: *ER_G* is defined as greenhouse gas emissions per unit of sales, *ER_E* is the energy consumption per unit of sales, *ER_GI* is the increase in greenhouse gas emissions per unit of sales, and *ER_EI* as the increase in energy consumption per unit of sales.

We include financial and non-financial factors that are expected to affect the credit rating in the model as control variables. The financial factors include firm size (*SIZE*), growth rate of sales (*GROW*), net profit margin (*ROA*), and debt-to-equity ratio (*LEV*) [38,39]. In general, firm size is the potential capability to have market power, the net asset margin indicates performance, and debt ratio represents default risk. Thus, *SIZE* and *ROA* are expected to have a positive effect on *GRADE*, whereas the *LEV* is expected to have a negative effect.

Further control variables included in the model are systemic risk (*BETA*), measured by a market beta coefficient [40,41], and foreign investor equity (*FOR*) and largest shareholders equity ratio (*LAR*), which control for the corporate governance structure. Dummy variables for audit firm’ size (*AUDIT*), KOSPI or not (*MKT*), industry (*IND*), and year (*YD*) are also included as additional control variables. To control heterogeneity by industry and time, we include industry at the 2-digit KSIC level and year fixed effects in the regression models [42,43]. Industry classification is based on the Korea Standard of Industry Classification (KSIC) section code. KSIC is divided into 21 sections and each section is denoted by a single letter from A to U.

(1) $$\begin{array}{cc} GRADE_{i,t+1}\; & =\;\beta_{1}ER_{i,\;t}\;+\;\beta_{2}SIZE_{i,\;t}\;+\;\beta_{3}GROW_{i,\;t}\;+\;\beta_{4}ROA_{i,\;t}\;+\;\beta_{5}LEV_{i,\;t}+\;\beta_{6}BETA_{i,\;t}\\ & \;+\;\beta_{7}FOR_{i,\;t}\;+\;\beta_{8}LAR_{i,\;t}+\beta_{9}AUDIT_{i,\;t}\;+\;\beta_{10}AV\_PMDA_{i,\;t}+\beta_{11}MKT_{i,\;t}\\ & \;+\sum IND\;+\;\sum YD\;+\;\varepsilon_{i,t} \end{array}$$

Equation (2) was used to test Hypothesis 2 regarding the moderating effect of market competition on the relationship between environmental risk and corporate credit ratings. In the regression equation that determines the credit rating score (*GRADE*), environmental risk (*ER*), market competition (*HHI*), and an interaction term (*ER* × *HHI*) are included to examine the moderating effect of market competition on the negative relationship between environmental risk and credit ratings. The higher the *HHI* (Herfindahl-Hirschman Index), which represents market competition, the lower the level of competition. Thus, if the coefficient β_3_ for *ER* × *HHI*, which represents environmental pollution firms with low market competition, is negative, it suggests that the negative effect of environmental risk on corporate credit ratings is strengthened in firms with low market competition.

(2) $$\begin{array}{cc} GRADE_{i,t+1}\; & =\;\beta_{1}ER_{i,\;t}\;+\;\beta_{2}HHI_{i,\;t}\;+\;\beta_{3}\left(ER_{i,\;t}\times HHI_{i,\;t}\right)\;+\;\beta_{4}SIZE_{i,\;t}\;+\;\beta_{5}GROW_{i,\;t}\\ & \;+\;\beta_{6}ROA_{i,\;t}\;+\;\beta_{7}LEV_{i,\;t}\;+\;\beta_{8}BETA_{i,\;t}+\beta_{9}FOR_{i,\;t}\;+\;\beta_{10}LAR_{i,\;t}\;+\;\beta_{11}AUDIT_{i,\;t}\\ & \;+\;\beta_{12}AV\_PMDA_{i,\;t}\;+\;\beta_{13}MKT_{i,\;t}\;+\;\sum IND\;+\;\sum YD\;+\;\varepsilon_{i,\;t} \end{array}$$

### 3.2. Sampling and Data Collection

This study’s sample includes firms listed on the Korea Stock Exchange (KSE) and Korean Securities Dealers Automated Quotations (KOSDAQ) for the fiscal years from 2011 to 2019, that reported energy use and greenhouse gas emissions to the Ministry of Environment according to ‘Management of Targets for Greenhouse Gas and Energy’. We obtained the credit rating information and financial data from the Data Guide (equivalent to Compustat and CRSP in the United States), and data on greenhouse gas emissions and energy consumption from the National Greenhouse Gas Management System (NGMS). The final sample consisted of 510 firm-years, after removing several observations for which financial and non-financial data were not available. Finally, we winsorized the main variables at the top and bottom 1% to minimize the effect of extreme variable values on the analysis results.

## 4. Results and Discussion

### 4.1. Descriptive Statistics and Correlation

Table 1 presents descriptive statistics for the variables used in this study. The mean of *GRADE* is approximately 15.247; thus, the average credit rating of the sample is therefore between A+ and A. The mean of *ER_G* and *ER_E* is approximately 77.635 and 0.810, respectively; thus, the average greenhouse gas emissions per KRW1 billion in sales is 77.635 tCO₂-eq, and the average energy consumption per KRW 1 billion in sales is 0.810 TJ. The mean and median *HHI* values were 0.291 and 0.241, respectively.

The mean of *SIZE* is 22.099, that of *GROW* is 0.058, that of *ROA* is 0.021, that of *LEV* is 1.926, and that of *BETA* is 0.946. The means of *FOR* and *LAR*, which are the governance (shareholder characteristic) variables, are 0.157 and 0.419, respectively; thus, the average foreign investor ownership and largest shareholder ownership are 15.7% and 41.9%, respectively. The proportion of firms serviced by Big4 audit firms is 93.4%.

Table 2 shows the classification matrix of actual credit ratings and expected credit ratings for 510 samples. Actual ratings are presented by row and expected ratings are presented by column. Expected ratings are defined as the rating level with the highest fitted probability from Equation (1). Thus, the row and column combination for the same rating level indicate predictive accuracy of the model. For example, the 29 firm-years with both A+ actual rating and A+ expected rating are correctly predicted cases.

Table 3 presents the Pearson correlation matrix for the key variables. *ER* is significantly and negatively associated with *GRADE*. This result implies that the lower the environmental risk, the higher the credit rating. Moreover, *HHI, SIZE*, *ROA*, *FOR*, and *AUDIT* are positively associated with *GRADE*, whereas *LEV* and *AV_PMDA* are negatively associated with *GRADE*. However, correlation analysis shows a simple correlation between variables, making it necessary to verify the hypothesis through multivariate regression analysis that considers the control variables that may affect the credit ratings.

### 4.2. Review of the Relationship between Environmental Risk and Credit Ratings

The results of testing Hypothesis 1 on the relationship between environmental risk and credit rating evaluations are presented in Table 4. This table presents the results of the ordered logit regression, which tests Hypothesis 1. We analyze each of the four regression models using the proxy of *ER* (*ER_G* or *ER_E* or *ER_GI* or *ER_EI*) as independent variables. We predict that environmental risk has a negative effect on the corporate bond credit ratings. Therefore, the coefficient of *ER* is expected to be significantly negative in each model.

The results presented in Table 4 support our hypotheses. In columns (1)–(4) of Table 4, the values of the coefficients of *ER* are −0.363, −0.315, −0.687, and −0.786, respectively, and significant at the 1%, 5%, 10%, and 10% levels (z-stat: −2.89, −1.98, −1.82, −1.77).

Thus, we find a negative association between environmental risk and bond credit ratings, controlling for the financial and non-financial factors that affect the credit rating. This result implies that CRAs may evaluate a firm’s environmental risk as a negative factor when evaluating credit ratings. Ultimately, we report that the more severe the firm’s environmental pollution, the higher the firm’s capital cost.

In particular, this study identified the effect of a firm’s greenhouse gas emissions and energy consumption, which are direct indicators of environmental risk, rather than CSR or ESG, on credit ratings. Many previous studies report that environmental risks caused by inefficient operation impair financial performance, such as firm value [12,13,14,15,16,17,18,19]. Thus, firms exposed to high environmental risk may have incentives to report high accounting performance through earnings management for green washing [44]. Therefore, this result has great significance in examining the effect of environmental risk, measured as direct environmental performance not affected by green washing, on credit rating.

The signs of the coefficients for *SIZE*, *ROA*, *FOR*, *LAR*, and *AUDIT* are significantly positive, but the sign of the coefficient for *LEV* is significantly negative. These results imply that the larger the firm size, the higher the return on assets, the higher the foreign investor equity ratio, the higher the largest shareholder equity ratio, if audited by Big4 audit firms, and the lower the debt ratio, the lower the credit rating.

Ordered logit regression is based on the parallel regression assumption, according to which the relationship between all pair of outcome groups is the same. To check for this hypothesis, we ran the Brant test [45]. We found that a significant test statistic provides evidence that the parallel regression assumption was violated. Specifically, the chi^2^ value of *SIZE*, *LEV*, and *BETA* was lower than the probability value (<0.05). Thus, we used the generalized ordered logit regression model, a form of unconstrained ordinal logit regression model, to relax the proportionality assumptions [46].

In the generalized ordered logit model with the qualitative dependent variable, two separate logit models were preferred because the dependent variable has three category levels. Here, we define the credit rating variable as a sequence variable divided into A, B, and C broad credit ratings for convenience of interpretation. Table 5 present results for the generalized ordered logit model method. In columns (1) and (2) of Table 5, the values of the coefficients of *ER_G* are −0.145 and −0.364, respectively, and significant at the 5% and 1% levels (z-stat: −2.02, −3.10). Thus, we find that, as the greenhouse gas emission increases, the probability of a credit rating of A/B is lower than that of C. In the same way, we know that, as the greenhouse gas emission increases, the probability of a credit rating of A is lower than that of B/C.

In addition, we analyzed the logit model using the credit rating dummy variable divided into investment grades (AAA~BBB−) and speculative grades (BB+~D). In the logit model, using a dummy variable of 1 if it is investment grade and 0 otherwise, as a dependent variable, the coefficient for *ER_G* is a significantly negative value. Thus, we know the higher the *ER_G*, the higher the probability of speculative grade. These results support Hypothesis 1 that environmental risk harms corporate bond credit ratings.

The Credit rating is a comprehensive indicator of credit risk that may capture corporate social responsibility (CSR) in the rating process. Here, CSR can cover environmental risk. Thus, we need to test the impact of environmental risk on credit risk by considering the level of CSR. First, we divided the whole sample into CSR firms and non-CSR firms, and then analyzed a regression model that examines the relationship between environmental risks and credit risk in each sample. In Korea, the criteria to divide CSR firms and non-CSR firms are as follows. The Korea Economic Justice Institute (KEJI), affiliated with the Citizens’ Coalition for Economic Justice, evaluates corporate social responsibility (CSR) activities of domestic listed firms every year and selects the top 200 firms. The reliability of the KEJI Index has been strengthened by its 20-year publication history and its widespread use in research and practice e.g., [47,48]. Based on this prior research, we defined firms included in KEJI Index ranking (top 200) as CSR firms, and those not included as non-CSR firms.

Table 6 presents a negative relationship between environmental risk and credit rating in the groups of both CSR firms and non-CSR firms. We find that environmental risk has a significant impact on the credit rating, even in the analysis considering the CSR propensity of firms. Therefore, Hypothesis 1 is strongly supported in the analysis considering the CSR level, which is a qualitative characteristic that is expected to be reflected in the credit rating evaluation.

It may be possible that environmental risk (*ER*) is not an exogenous variable but an endogenous variable derived according to a firm’s asset structure and business performance. Thus, we used a two-stage least squares (2SLS) approach to verify the robustness of the effect of environmental risk on corporate bond ratings.

In the first stage, we searched for instrumental variables (IV), as factors that have no correlation with the error term or impact on *ER*. The instrument variables were selected based on the over-identification test. The main advantage of IV is that we are explicit about the causes of variation used to evaluate the relative effect of environmental risk on credit rating. We used the ratio of tangible assets to total assets (*TR*) as an instrument for the magnitude of environmental risk. This is because production activities with facility assets generate energy consumption, resulting in pollutant emissions [49]. This also mitigates concerns about the causal interpretation of the results, as variation of total asset ratio is orthogonal to firm’s performance [50]. In the second stage, the endogenous variable is replaced with its predicted value from the first-stage estimation before being regressed on the variables. Through this, we can see the results of regression analysis in which the possibility of endogeneity is controlled.

Table 7 reports instrumental-variables two-stage least-squares (IV-2SLS) results. Column (1) presents the first-stage estimation results of the instrument variable, *TR*, which is related to *ER_G*. The sign of the coefficient for *TR* is significantly positive; thus, we know that the higher the concentration of tangible assets, the greater the environmental risk. The signs of coefficients for *SIZE, FOR, AUDIT*, and *MKT* are significantly negative. These results indicate that the larger the firm size, the higher the foreign investor equity ratio, if audited by Big4 audit firms, and if a firm belongs to the KOSPI market, the lower the environmental risk. Column (2) reports the second-stage estimation results for hypothesis testing. The coefficient of *ER_G* is negative (*t*-stat: −3.17) and significant (*p* < 0.01). This finding is consistent with the negative effect of environmental risk on corporate bond credit ratings. Therefore, we know that Hypothesis 1, i.e., environmental risk harms corporate bond credit ratings, is strongly supported in 2SLS.

### 4.3. Review of the Moderating Effects of Market Competition on the Relationship between Environmental Risk and Credit Ratings

Table 8 shows the test results of the moderating effects of market competition on the relationship between environmental risk and credit ratings. The negative relationship between environmental risk and corporate bond credit ratings is expected to be stronger for firms with low market competition in Hypothesis 2. Therefore, the coefficient of *ER* × *HHI* is expected to be significantly negative. In columns (1)–(4) of Table 8, the values of the coefficients of *ER* × *HHI* are −0.272, −0.229, −0.373, and −0.250, respectively, and significant at the 5%, 10%, 1%, and 10% levels (z-stat: −2.23, −1.84, −2.99, −1.69). Thus, Hypothesis 2 is supported. This result means that the negative effect of environmental risk on corporate bond credit ratings is stronger in firms with low market competition. Thus, we suggest that market competition has an important moderating effect on the relationship between environmental risk and credit ratings.

To verify the robustness of the moderating effects of market competition between environmental risk and corporate bond credit ratings, we analyzed Hypothesis 2 using a dummy variable of *HHI* based on the regulations of the Korea Fair Trade Commission (FTC). The Fair Trade Commission (FTC) uses *HHI* as a standard in the examination of business combinations, which is classified into fewer than 1200 (non-concentrated market), 1200–2500 (slightly concentrated market), and more than 2500 (very concentrated market). A dummy variable is defined based on whether the *HHI* value as the market competition index exceeds 0.25. We estimate that if *HHI* is greater than 0.25, market competition is low, and if *HHI* is less than 0.25, market competition is high. That is, *HHI_D* is a dummy variable that is 1 if *HHI* is greater than 0.25, and 0 otherwise.

Table 9 shows the test results for the moderating effects of market competition using *HHI_D* as a proxy. The coefficient of *ER × HHI* is expected to be significantly negative. In columns (1)–(4) of Table 9, the values of the coefficients of *ER* × *HHI* are −1.548, −0.025, −0.209, and −0.136, respectively, and significant at the 5%, 10%, 10%, and 10% levels (z-stat: −2.44, −1.79, −1.70, −1.69). This result is consistent with Table 8, and thus Hypothesis 2 is strongly supported.

## 5. Conclusions

We investigated the relationship between environmental risk and corporate bond credit ratings, and the moderating effect of market competition. Our results show a negative association between environmental risk and bond credit ratings. This result implies that credit rating agencies (CRAs) may evaluate a firm’s environmental risk as a negative factor when evaluating credit ratings. Ultimately, we think that the more severe the environmental pollution, the higher the firm’s capital cost. We also find that the negative effect of environmental risk on corporate bond credit ratings is stronger in firms with low market competition. This result means that the negative effect of environmental risk on corporate bond credit ratings is stronger in firms with low market competition. Thus, we suggest that market competition has an important moderating effect on the relationship between environmental risk and credit ratings.

This study contributes to prior research on corporate bond ratings by improving our understanding of the effect of environmental risk on credit ratings. As environmental risks impair financial performance, such as firm value, firms exposed to high environmental risk may have incentives to report strong accounting performance through earnings management for green washing. Thus, it is significant to examine the effect of environmental risk, measured as direct environmental performance not affected by green washing, on credit rating. Therefore, we provide a convincing answer to the puzzle in environmental management, beyond the determinants of credit rating, which have been discussed in previous studies. We also suggest that policymakers need to manage market competition in terms of environmental justice, given that market competition has a significant moderating effect on the relationship between environmental risk and credit ratings. Sound competition can limit the risk environmental problems in the capital market by inducing efficient management of firms. Therefore, it is necessary to evaluate and control the degree of market competition by industry to control firms’ environmental risk at the policy level.

However, there may be some difficulties in generalizing the results because this study targeted firms that have reported greenhouse gas emissions and energy consumption to the Ministry of Environment. In addition to market competition, we think that meaningful research is needed in the future on the role of stakeholders who can influence a firm’s climate risk management, in terms of factors such as corporate governance, business strategy, and managerial characteristics.

## Figures and Tables

**Table 1:** Descriptive statistics.

Variable		Average	Standard Deviation	Min	Median	Max
*GRADE*		15.247	3.162	4.000	16.000	19.000
Environmental Risk	*ER_G*	0.338	0.838	0.004	0.087	6.526
	*ER_E*	0.445	0.613	0.004	0.170	3.253
	*ER_GI*	−0.004	0.082	−0.522	−0.001	0.281
	*ER_EI*	−0.008	0.102	−0.666	−0.001	0.194
*HHI*		0.291	0.164	0.082	0.241	0.945
*SIZE*		22.099	1.428	19.157	21.900	25.368
*GROW*		0.058	0.222	−0.333	0.029	1.504
*ROA*		0.021	0.048	−0.168	0.025	0.140
*LEV*		1.926	2.582	0.196	1.340	20.126
*BETA*		0.946	0.400	0.000	0.931	1.839
*FOR*		0.157	0.124	0.003	0.131	0.548
*LAR*		0.419	0.142	0.118	0.391	0.713
*AUDIT*		0.934	0.249	0.000	1.000	1.000
*AV_PMDA*		0.038	0.031	0.000	0.031	0.172
*MKT*		0.968	0.176	0.000	1.000	1.000

Note. Variable definitions: *GRADE* = conversion of the letter ratings of KR, NICE, and KIS to a single numeric scale: AAA = 20, AA+ = 19, …, D = 1; *ER_G* = greenhouse gas emission per unit of sales (KRW million); *ER_E* = energy consumption per unit of sales (KRW 10 thousand); *ER_GI* = increase in greenhouse gas emissions per unit of sales; *ER_EI* = increase in energy consumption per unit of sales; *HHI* = Hirschman-Herfindahl Index; *SIZE* = firm size (natural logarithm of total assets); *GROW* = sales growth rate {(t term sales − t term 1 sales)/t term 1 sales}; *ROA* = return on assets (net earnings/total assets); *LEV* = debt to asset ratio (debt/net assets); *BETA* = systemic market risk; *FOR* = foreign investor equity ratio; *LAR* = largest shareholders equity ratio; *AUDIT* = indicator variable that takes the value 1 if a firm is audited by one of Big4 audit firms, and 0 otherwise; *AV_PMDA* = absolute value of performance matched discretionary accruals; *MKT* = indicator variable that takes the value 1 if a firm belongs to KOSPI market, and 0 otherwise.

**Table 2:** Actual credit ratings and expected credit rating classification matrix.

	AAA	AA+	AA	AA−	A+	A	A−	BBB+	BBB	BBB−	BB+	BB	BB−	B+	B	B−	CCC	CC	D
AAA	9	5	4	2															
AA+	8	36	21	1	1		2	1											
AA	2	8	22	14	9	2	2				1								
AA−			4	17	19	8	7	1		1			1						
A+			2	9	29	25	1	2		1									
A			3	3	7	23	13	3	1		1								
A−		1		2	4	16	26	25	1										
BBB+			1			3	7	8	4	2			1						
BBB				1		2	3	7	9	2									
BBB−							2	2	8	5	1								
BB+									1	1	2		1						
BB									2	1	1	2							
BB−							1		1		1	3	1	1					
B+												1	1	1					
B														2					
B−														1	1	2			
CCC														2	3			1	
CC																1		1	
D																			

Note. The value is the number of samples (firm-years) that match the actual credit rating and the expected credit rating.

**Table 3:** Pearson correlations.

	*GRADE*	*ER_G*	*ER_E*	*ER_GI*	*ER_EI*	*HHI*	*SIZE*	*GROW*	*ROA*	*LEV*	*BETA*	*FOR*	*LAR*	*AUDIT*	*AV* *_PMDA*
*GRADE*	1														
*ER_G*	−0.19 ***	1													
*ER_E*	−0.26 ***	0.80 ***	1												
*ER_GI*	−0.16 ***	0.06	0.12 **	1											
*ER_EI*	−0.17 ***	0.07	0.12 **	0.77 ***	1										
*HHI*	0.33 ***	−0.16	−0.03	0.02	−0.06 *	1									
*SIZE*	0.47 ***	−0.17 ***	−0.34 ***	−0.09 *	−0.09 *	0.36 ***	1								
*GROW*	0.04	0.00	0.00	−0.14 **	−0.14 ***	−0.01	0.01	1							
*ROA*	0.35 ***	−0.06	−0.07	−0.08	−0.07	0.01	0.06	0.03	1						
*LEV*	−0.33 ***	0.01	−0.01	−0.01	0.02	−0.09	−0.03	−0.01	−0.25 ***	1					
*BETA*	0.01	−0.03	−0.03	0.15 ***	0.18 ***	−0.02	0.34 ***	−0.04	−0.09 *	0.02	1				
*FOR*	0.53 ***	−0.07	−0.19 ***	−0.17 ***	−0.14 ***	0.26 ***	0.53 ***	−0.01	0.26 ***	−0.14 ***	0.11 **	1			
*LAR*	0.07	−0.03	0.07	0.02	−0.03	0.05	−0.15 ***	0.02	0.13 ***	0.06	−0.11 **	−0.25 ***	1		
*AUDIT*	0.25 ***	−0.24 ***	−0.17 ***	−0.18 ***	−0.15 ***	0.10 ***	0.22 ***	0.03	0.12 **	0.07	−0.00	0.16 ***	0.06	1	
*AV_PMDA*	−0.24 ***	0.08 *	0.13 ***	0.01	0.05	−0.08 **	−0.09 *	0.09 *	−0.41 ***	0.15 ***	0.01	−0.04	−0.18 ***	−0.05	1
*MKT*	0.06	0.01	−0.03	−0.12 **	−0.12 **	0.08 **	0.17 ***	0.01	−0.06	0.09 *	0.14 ***	0.16 ***	0.09 *	0.06	−0.08

Note. *, **, and *** denote significance at the 10%, 5%, and 1% levels, respectively and detailed definitions of variables are in the notes of Table 1.

**Table 4:** Effect of environmental risk on corporate bond credit ratings (H1).

$\begin{array}{cc} GRADE_{i,t+1}\; & =\;\beta_{1}ER_{i,\;t}\;+\;\beta_{2}SIZE_{i,\;t}\;+\;\beta_{3}GROW_{i,\;t}\;+\;\beta_{4}ROA_{i,\;t}\;+\;\beta_{5}LEV_{i,\;t}+\;\beta_{6}BETA_{i,\;t}\\ & \;+\;\beta_{7}FOR_{i,\;t}+\;\beta_{8}LAR_{i,\;t}+\beta_{9}AUDIT_{i,\;t}\;+\;\beta_{10}AV\_PMDA_{i,\;t}+\beta_{11}MKT_{i,\;t}\\ & \;+\sum IND\;+\;\sum YD\;+\;\varepsilon_{i,\;t} \end{array}$				
Variable	*ER_G*	*ER_E*	*ER_GI*	*ER_EI*
	Coef. (z-Value)	Coef. (z-Value)	Coef. (z-Value)	Coef. (z-Value)
*ER*	−0.363 (−2.89) ***	−0.315 (−1.98) **	−0.687 (−1.82) *	−0.786 (−1.77) *
*SIZE*	1.148 (8.50) ***	0.882 (7.88) ***	1.079 (6.82) ***	1.060 (6.89) ***
*GROW*	0.069 (0.70)	0.067 (0.68)	−0.209 (−1.06)	−0.424 (−0.90)
*ROA*	5.126 (3.36) ***	10.62 (3.73) ***	7.615 (2.94) ***	10.277 (3.03) ***
*LEV*	−0.304 (−5.20) ***	−0.304 (−5.18) ***	−0.189 (−4.48) ***	−0.187 (−4.80) ***
*BETA*	0.440 (1.29)	0.370 (1.08)	0.523 (1.34)	0.525 (1.35)
*FOR*	9.868 (6.76) ***	9.406 (6.48) ***	12.338 (6.46) ***	12.241 (6.60) ***
*LAR*	5.230 (6.05) ***	3.543 (4.52) ***	5.501 (5.87) ***	5.57 (5.92) ***
*AUDIT*	2.646 (5.85) ***	1.347 (5.73) ***	2.466 (4.94) ***	2.39 (5.03) **
*AV_PMDA*	3.754 (1.01)	4.485 (1.24)	2.985 (0.65)	3.980 (0.90)
*MKT*	0.114 (0.17)	0.229 (0.35)	0.898 (0.96)	0.933 (1.01)
Industry Fixed Effect	Yes	Yes	Yes	Yes
Year Fixed Effect	Yes	Yes	Yes	Yes
Pseudo R^2^	0.35	0.34	0.35	0.35
No. of Obs.	510	510	510	510

Note. *, **, and *** denote significance at the 10%, 5%, and 1% levels, respectively and detailed definitions of variables are in the notes of Table 1.

**Table 5:** Generalized ordered logit model estimations.

$\begin{array}{cc} GRADE_{i,t+1}\; & =\;\beta_{1}ER\_G_{i,\;t}\;+\;\beta_{2}SIZE_{i,\;t}\;+\;\beta_{3}GROW_{i,\;t}\;+\;\beta_{4}ROA_{i,\;t}\;+\;\beta_{5}LEV_{i,\;t}\;+\;\beta_{6}BETA_{i,\;t}\\ & \;+\;\beta_{7}FOR_{i,\;t}\;+\;\beta_{8}LAR_{i,\;t}+\beta_{9}AUDIT_{i,\;t}\;+\;\beta_{10}AV_{\_}PMDA_{i,\;t}+\beta_{11}MKT_{i,\;t}\\ & \;+\sum IND\;+\;\sum YD\;+\;\varepsilon_{i,\;t} \end{array}$				
Variable	Grade A, B vs. Grade C		Grade A vs. Grade B, C	
	Coef.	(z-Value)	Coef.	(z-Value)
*ER_G*	−0.145	(−2.02) **	−0.364	(−3.10) ***
Control Variables	Yes	Yes	Yes	Yes
Industry Fixed Effect	Yes	Yes	Yes	Yes
Year Fixed Effect	Yes	Yes	Yes	Yes
Pseudo R^2^	0.35	0.34	0.35	0.35
No. of Obs.	510	510	510	510

Note. **, and *** denote significance at the 10%, 5%, and 1% levels, respectively and detailed definitions of variables are in the notes of Table 1.

**Table 6:** Effect of environmental risk on credit ratings (CSR firms vs. non-CSR firms).

$\begin{array}{cc} GRADE_{i,t+1}\; & =\;\beta_{1}ER_{i,\;t}\;+\;\beta_{2}SIZE_{i,\;t}\;+\;\beta_{3}GROW_{i,\;t}\;+\;\beta_{4}ROA_{i,\;t}\;+\;\beta_{5}LEV_{i,\;t}+\;\beta_{6}BETA_{i,\;t}\\ & \;+\;\beta_{7}FOR_{i,\;t}\;+\;\beta_{8}LAR_{i,\;t}+\beta_{9}AUDIT_{i,\;t}\;+\;\beta_{10}AV\_PMDA_{i,\;t}+\beta_{11}MKT_{i,t}\\ & \;+\sum IND\;+\;\sum YD\;+\;\varepsilon_{i,\;t} \end{array}$				
CSR Firms				
Variable	*ER_G*	*ER_E*	*ER_GI*	*ER_EI*
	Coef. (z-Value)	Coef. (z-Value)	Coef. (z-Value)	Coef. (z-Value)
*ER*	−0.762 (−2.91) ***	−0.912 (−1.99) **	−0.758 (−1.98) **	−0.884 (−1.79) *
Control Variables	Yes	Yes	Yes	Yes
Industry Fixed Effect	Yes	Yes	Yes	Yes
Year Fixed Effect	Yes	Yes	Yes	Yes
Pseudo R^2^	0.39	0.39	0.38	0.38
No. of Obs.	241	241	241	241
Non-CSR firms				
Variable	*ER_G*	*ER_E*	*ER_GI*	*ER_EI*
	Coef. (z-value)	Coef. (z-value)	Coef. (z-value)	Coef. (z-value)
*ER*	−0.690 (−2.59) ***	−0.861 (−1.69) *	−0.289 (−1.70) *	−0.445 (−1.96) *
Control Variables	Yes	Yes	Yes	Yes
Industry Fixed Effect	Yes	Yes	Yes	Yes
Year Fixed Effect	Yes	Yes	Yes	Yes
Pseudo R^2^	0.35	0.34	0.35	0.35
No. of Obs.	269	269	269	269

Note. *, **, and *** denote significance at the 10%, 5%, and 1% levels, respectively and detailed definitions of variables are in the notes of Table 1.

**Table 7:** Robustness test of hypothesis 1 by 2SLS.

$\begin{array}{c} {}[1\;\mathrm{Stage}]\;ER_{i,\;t}\;=\;\beta_{0}\;+\beta_{1}TR_{i,\;t}\;+\;\beta_{2}SIZE\;+\beta_{3}GROW_{i,\;t}\;+\;\beta_{4}ROA_{i,\;t}\;+\;\beta_{5}LEV_{i,\;t}\;+\;\beta_{6}BETA_{i,\;t}\\ \;\;\;\;\;\;\;\;\;\;\;\;\;\;\;\;\;\;\;\;\;\;\;\;\;\;\;\;\;\;\;\;\;\;\;\;\;\;\;+\;\;\beta 7FOR_{i,\;t}\;+\;{\;\beta }_{8}LAR_{i,\;t}\;+\;\beta_{9}AUDIT_{i,\;t}\;+\;\beta_{10}AV\_PMDA_{i,\;t}\;+\;\beta_{11}MKT_{i,\;t}\\ \;\;\;\;\;\;\;\;\;\;\;\;\;\;\;\;\;\;\;\;\;\;\;\;\;\;\;\;\;\;\;\;\;\;\;\;\;\;\;+\;\sum IND\;+\;\sum YD\;+\;\varepsilon_{i,\;t}\\ {}[2\;\mathrm{Stage}]\;GRADE_{i,\;t+1}\;=\;\beta_{0}\;+\;\beta_{1}{\hat{ER}}_{i,t}\;+\;\beta_{2}SIZE_{i,\;t}\;+\;\beta_{3}GROW_{i,\;t}\;+\;\beta_{4}ROA_{i,\;t}\;+\;\beta_{5}LEV_{i,\;t}\\ \;\;\;\;\;\;\;\;\;\;\;\;\;\;\;\;\;\;\;\;\;\;\;\;\;\;\;\;\;\;\;\;\;\;\;\;\;\;\;\;\;\;\;\;\;\;\;\;\;\;\;\;+\;{\;\beta }_{6}BETA_{i,\;t}\;+\;\beta_{7}FOR_{i,\;t}\;+\;\beta_{8}LAR_{i,\;t}\;+\;\beta_{9}AUDIT_{i,\;t}\;+\;\beta_{10}AV\_PMDA_{i,\;t}\\ \;\;\;\;\;\;\;\;\;\;\;\;\;\;\;\;\;\;\;\;\;\;\;\;\;\;\;\;\;\;\;\;\;\;\;\;\;\;\;\;\;\;\;\;\;\;\;\;\;\;\;\;+\;{\;\beta }_{11}MKT_{i,\;t}\;+\;\sum IND\;+\;\sum YD\;+\;\varepsilon_{i,\;t} \end{array}$				
Variable	First Stage Regression		IV Regression	
	Coef.	*t*-Value	Coef.	*t*-Value
*TR*	2.118	(5.84) ***		
$\hat{ER}$			−1.333	(−3.17) ***
*SIZE*	−0.202	(−4.25) ***	0.764	(4.00) ***
*GROW*	0.032	(0.52)	0.132	(0.90)
*ROA*	0.537	(0.56)	2.339	(1.05)
*LEV*	−0.006	(−0.28)	−0.323	(−8.59) ***
*BETA*	0.285	(1.93) *	−0.361	(−1.13)
*FOR*	−1.024	(−2.24) **	11.458	(9.45) ***
*LAR*	−0.023	(−0.10)	3.699	(4.23) ***
*AUDIT*	−0.513	(−3.10) ***	0.808	(1.25)
*AV_PMDA*	−1.105	(−1.40)	4.227	(0.96)
*MKT*	−1.111	(−4.08) ***	−0.755	(−0.89)
Intercept	1.328	(4.52) ***	1.929	(3.42) ***
Industry Fixed Effect	Yes		Yes	
Year Fixed Effect	Yes		Yes	
Adjusted R^2^	0.18		0.45	
No. of Obs.	510		510	

Note. *, **, and *** denote significance at the 10%, 5%, and 1% levels, respectively. *TR* is the ratio of tangible assets to total assets, and *ER* uses *ER_G* as a representative variable. Detailed definitions of the other variables are in the notes of Table 1.

**Table 8:** Moderating effect of market competition (H2).

$\begin{array}{cc} GRADE_{i,t+1}\; & =\;\beta_{1}ER_{i,\;t}\;+\;\beta_{2}HHI_{i,\;t}\;+\;\beta_{3}\left(ER_{i,\;t}\times HHI_{i,\;t}\right)\;+\;\beta_{4}SIZE_{i,\;t}\;+\;\beta_{5}GROW_{i,\;t}\;+\;\beta_{6}ROA_{i,\;t}\\ & \;+\;\beta_{7}LEV_{i,\;t}\;+\;\beta_{8}BETA_{i,\;t}+\beta_{9}FOR_{i,\;t}\;+\;\beta_{10}LAR_{i,\;t}\;+\;\beta_{11}AUDIT_{i,\;t}\;+\;\beta_{12}AV\_PMDA_{i,\;t}\\ & \;+\;\beta_{13}MKT_{i,\;t}\;+\;\sum IND\;+\;\sum YD\;+\;\varepsilon_{i,\;t} \end{array}$				
Variable	*ER_G*	*ER_E*	*ER_GI*	*ER_EI*
	Coef. (z-Value)	Coef. (z-Value)	Coef. (z-Value)	Coef. (z-Value)
*ER*	−0.494 (−1.31)	−0.266 (−0.65) **	0.414 (0.14)	−0.454 (−0.60)
*HHI*	−0.437 (−1.86) *	−1.089 (−0.77)	0.547 (0.14)	−0.532 (−1.38)
*ER* × *HHI*	−0.272 (−2.23) **	−0.229 (−1.84) *	−0.373 (−2.99) ***	−0.250 (−1.69) *
*SIZE*	1.179 (8.45) ***	1.003 (7.42) ***	0.806 (7.00) ***	0.823 (6.35) ***
*GROW*	0.127 (1.26)	0.068 (0.69)	0.537 (0.22)	0.224 (0.32)
*ROA*	9.388 (3.28) ***	9.201 (3.25) **	7.127 (2.23) **	8.913 (2.70) ***
*LEV*	−0.297 (−5.13) ***	−0.298 (−5.06) ***	−0.262 (−4.70) ***	−0.247 (−4.44) ***
*BETA*	0.118 (0.32)	0.394 (1.22)	0.321 (0.78)	−1.167 (−3.46) ***
*FOR*	9.208 (7.10) ***	9.379 (6.80) ***	9.314 (8.17) ***	9.129 (4.79) ***
*LAR*	5.184 (5.88) ***	5.166 (5.93) ***	4.174 (4.87) ***	4.129 (4.79) ***
*AUDIT*	2.890 (6.21) ***	2.054 (4.64) ***	2.346 (3.07) ***	1.458 (3.35) ***
*AV_PMDA*	3.168 (0.85)	3.655 (0.49)	2.942 (0.65)	3.926 (1.40)
*MKT*	0.289 (0.44)	0.354 (0.59)	0.805 (0.85)	0.723 (0.77)
Industry Fixed Effect	Yes	Yes	Yes	Yes
Year Fixed Effect	Yes	Yes	Yes	Yes
Pseudo R^2^	0.36	0.35	0.36	0.36
No. of Obs.	510	510	510	510

Note. *, **, and *** denote significance at the 10%, 5%, and 1% levels, respectively and detailed definitions of variables are in the notes of Table 1.

**Table 9:** Robustness test of Hypothesis 2 by HHI based on the regulations of the Korea FTC.

$\begin{array}{cc} GRADE_{i,\;t+1}\; & =\;\beta_{1}ER_{i,\;t}\;+\;\beta_{2}HHI_{-}D_{i,\;t}\;+\;\beta_{3}\left(ER_{i,\;t}\times HHI_{i,\;t}\right)\;+\;\beta_{4}SIZE_{i,\;t}\;+\;\beta_{5}GROW_{i,\;t}\\ & \;+\;\beta_{6}ROA_{i,\;t}\;+\;\beta_{7}LEV_{i,\;t}\;+\;\beta_{8}BETA_{i,\;t}+\beta_{9}FOR_{i,\;t}\;+\;\beta_{10}LAR_{i,\;t}\;+\;\beta_{11}AUDIT_{i,\;t}\\ & \;+\;\beta_{12}AV_{-}PMDA_{i,\;t}\;+\;\beta_{13}MKT_{i,\;t}\;+\;\sum IND\;+\;\sum YD\;+\;\varepsilon_{i,\;t} \end{array}$				
Variable	*ER_G*	*ER_E*	*ER_GI*	*ER_EI*
	Coef. (z-Value)	Coef. (z-Value)	Coef. (z-Value)	Coef. (z-Value)
*ER*	0.249 (1.04)	−0.308 (−1.55)	−0.263 (−1.79) *	−0.731 (−1.82) *
*HHI_D*	−1.242 (−4.13) ***	−0.672 (−2.23) **	−0.322 (−1.86) *	−0.773 (−2.67) ***
*ER*×*HHI_D*	−1.548 (−2.44) **	−0.025 (−1.79) *	−0.209 (−1.70) *	−0.136 (−1.69) *
Control Variables	Included	Included	Included	Included
Industry Fixed Effect	Yes	Yes	Yes	Yes
Year Fixed Effect	Yes	Yes	Yes	Yes
Pseudo R^2^	0.35	0.35	0.35	0.35
No. of Obs.	510	510	510	510

Note. *, **, and *** denote significance at the 10%, 5%, and 1% levels, respectively and detailed definitions of variables are in the notes of Table 1.

## Data Availability

Not applicable.
